# Mesenchymal stem cells prevent overwhelming inflammation and reduce infection severity via recruiting CXCR3^+^ regulatory T cells

**DOI:** 10.1002/cti2.1181

**Published:** 2020-09-30

**Authors:** Wenchao Li, Weiwei Chen, Saisai Huang, Genhong Yao, Xiaojun Tang, Lingyun Sun

**Affiliations:** ^1^ Department of Rheumatology and Immunology The Affiliated Drum Tower Hospital of Nanjing University Medical School Nanjing China

**Keywords:** autoimmune disease, CXCR3, mesenchymal stem cells, respiratory infection, Treg cells

## Abstract

**Objectives:**

Mesenchymal stem cells (MSCs) have shown great potential in treating autoimmune diseases (ADs). Unlike the traditional immunosuppressants, which inadvertently impair patients' antimicrobial immunity, MSCs reduce the incidence and duration of respiratory infection. However, the underlying mechanisms are unknown.

**Methods:**

To investigate how MSCs regulate the lung immunity and improve the defence against respiratory infection, we infected MSC‐treated wild‐type and lupus‐prone mice with *Haemophilus influenzae* intranasally and determined the clearance of bacteria. Tissue damage and inflammatory cytokines were measured by H&E staining and ELISA separately. Immune cell subsets in the tissues were analysed by flow cytometry.

**Results:**

MSC pretreatment prevented overwhelming inflammation and accelerated bacterial clearance in both wild‐type and lupus‐prone mice. Tregs increased dramatically in the lung after MSC treatment. Adoptive transfer of Tregs isolated from MSC‐treated mice offered similar protection, while deletion of Tregs abrogated the protective effects of MSCs. The majority of the intravenously injected MSCs were engulfed by lung phagocytes, which in turn produced CXCL9 and CXCL10 and recruited tremendous CXCR3^+^ Tregs into the lung. Compared with their CXCR3^−^ counterparts, CXCR3^+^ Tregs displayed enhanced proliferation and stronger inhibitory functions. Neutralisation of CXCL9 and CXCL10 significantly downregulated the migration of CXCR3^+^ Tregs and eliminated the benefits of MSC pretreatment.

**Conclusion:**

Here, we showed that by recruiting CXCR3^+^ Tregs, MSC treatment restricted the overactivation of inflammatory responses and prevented severe symptoms caused by infection. By discovering this novel property of MSCs, our study sheds light on optimising long‐term immunosuppressive regimen for autoimmune diseases and other immune disorders.

## Graphical abstract

Unlike traditional immunosuppressants, which inadvertently impair a patient's antimicrobial immunity during therapy, mesenchymal stem cells (MSCs) reduce the incidence and duration of respiratory infection. MSCs are phagocytosed by lung cells and recruit CXCR3^+^ Tregs which could prevent overwhelming inflammation and accelerate bacterial clearance. Our study highlights the application of MSCs in treating patients who need a long‐term immunosuppressive regimen.

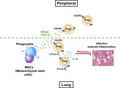

## Introduction

Mesenchymal stem cells (MSCs) are nonhaematopoietic stem cells, which are able to differentiate into a variety of cell types, including osteocytes, chondrocytes and adipocytes. They can be isolated from almost every tissue, such as bone marrow and umbilical cord.^1^, ^2^ Except for the application in tissue repair and regeneration, their inimitable immune regulatory properties make them an attractive therapeutic option for patients with chronic inflammation and autoimmune diseases (ADs).^3^ MSCs improve the disease outcomes either through paracrine secretion of multiple cytokines and cargo‐bearing exosomes or promoting the emergence and/or recruitment of regulatory/suppressive immune cell subsets, including regulatory T lymphocytes and alternatively activated (M2) macrophages.^4^

General immunosuppressant agents suppress not only the autoimmune responses but also the immunodefence against infection. As a consequence, microbial infection, especially in the respiratory tract, significantly increases in the AD patients and becomes a leading cause of death.^5^ It has been estimated that infection accounts for one‐third of deaths in the systemic lupus erythematosus (SLE) population.^6^, ^7^ However, for MSCs, one retrospective study reveals significant reduction, compared to before infusion, in the incidence and/or severity of respiratory infection in 7.7% of the enrolled SLE patients receiving treatment.^8^ This result is surprising and counterintuitive, since as aforementioned MSCs downregulate the host's immune responses. It is not known how MSC treatment leads to better control of the following respiratory infections. Elucidating the underlying mechanism will help us further optimise MSC therapy and benefit the patients who otherwise need long‐term immunosuppressive regimen.

*Haemophilus influenzae* (Hi) colonises in the human upper respiratory tract. When the host immunity is compromised, it can disseminate into the privileged anatomical locations and causes a wide spectrum of diseases such as pneumonia.^9^ It has been reported that Hi is frequently isolated from patients receiving immunosuppressive treatment.^10^ In this study, we employed the murine model of respiratory Hi infection to determine whether MSC pretreatment helps to avoid the development of clinical illness caused by infection and examined the underlying mechanisms.

## Results

### MSC pretreatment provides long‐term protection against the following pulmonary infection

**Figure 1:**
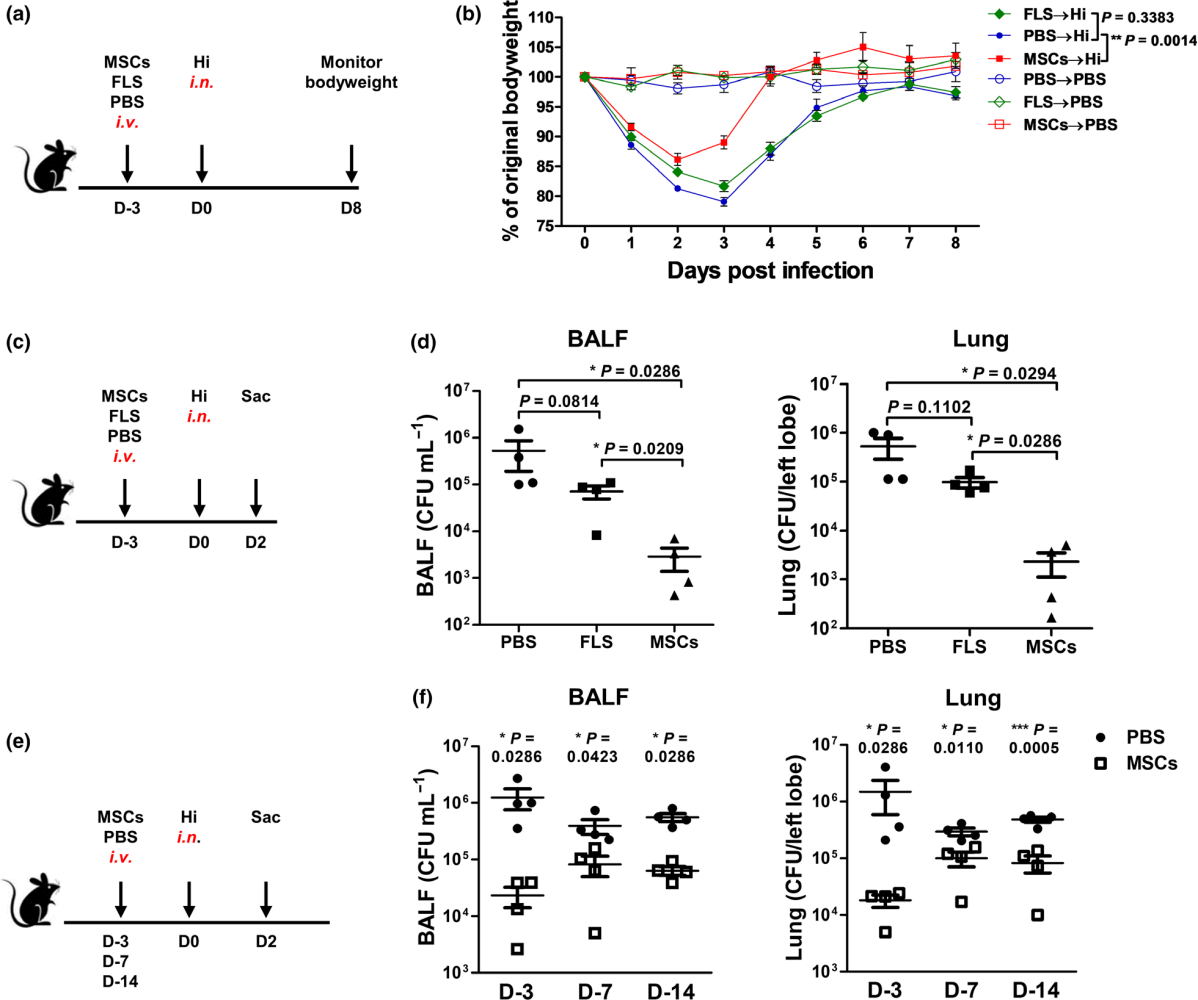
MSC pretreatment provides long‐term protection against the following pulmonary infection. **(a)** Scheme of the experiment. After receiving 5 × 10^5^ MSCs or fibroblasts (FLS) or PBS (*i.v*.), mice were infected with 1 × 10^8^ CFU Hi (*i.n*.). **(b)** Body weight changes were monitored. Uninfected mice were set as controls. n = 4 or 5 per group. **(c, d)** Mice were sacrificed on day 2 postinfection and bacteria in the BALF and lungs were determined. n = 4 per group. **(e, f)** Mice were treated with MSCs or PBS and then were infected with Hi at indicated time. Bacteria in the lungs were determined 2 days later. n = 4 per group. All the experiments were repeated at least three times. The representative data are shown.

Previous studies revealed that most of the intravenously administered MSCs ended up in the microvasculature of lung and disappeared rapidly within 24 h, suggesting that they exert long‐term therapeutic effects mainly by modulating the recipient's immune system.^11^, ^12^ Nevertheless, MSCs have also been discovered to kill bacteria directly.^13^, ^14^ In this study, we wanted to focus on how the MSC‐modulated immunity responses to the following infection. Thus, to avoid the potential interference by MSCs themselves, we infected C57BL/6 mice on day 3 after cell infusion, when nearly all the infused cells were cleared out of the lung (Supplementary figure 1). Mice were injected with 5 × 10^5^ MSCs via the intravenous route. Negative controls received 5 × 10^5^ synovial fibroblasts (FLS) or vehicle (PBS). After intranasal infection with 1 × 10^8^ CFU Hi, survival and body weight changes of the mice were monitored until they totally recovered (Figure 1a). Although all the mice survived from Hi infection, those from MSC‐treated group had less weight loss and recovered more quickly (Figure 1b). We next determined bacterial elimination by the mice. On day 2 after infection, bacteria in the bronchial alveolar lavage fluid (BALF) and lung were counted (Figure 1c). The results showed that PBS‐ and FLS‐pretreated mice had similar numbers of Hi, whereas those received MSCs had 100‐fold fewer bacteria (Figure 1d). Having confirmed the protective effect of MSCs, we then investigated that how long it could last. After infusing MSCs, mice were infected at the indicated time (Figure 1e). The data indicated that protection sustained more than 2 weeks, as mice treated with MSCs 14 days ago still had about 10‐fold fewer bacteria than the controls (Figure 1f). These data suggest that MSC‐modulated host immunity provides better protection against following pulmonary infection.

### MSC‐preconditioned lung restricts infection‐induced inflammation and allows for better bacterial elimination

**Figure 2:**
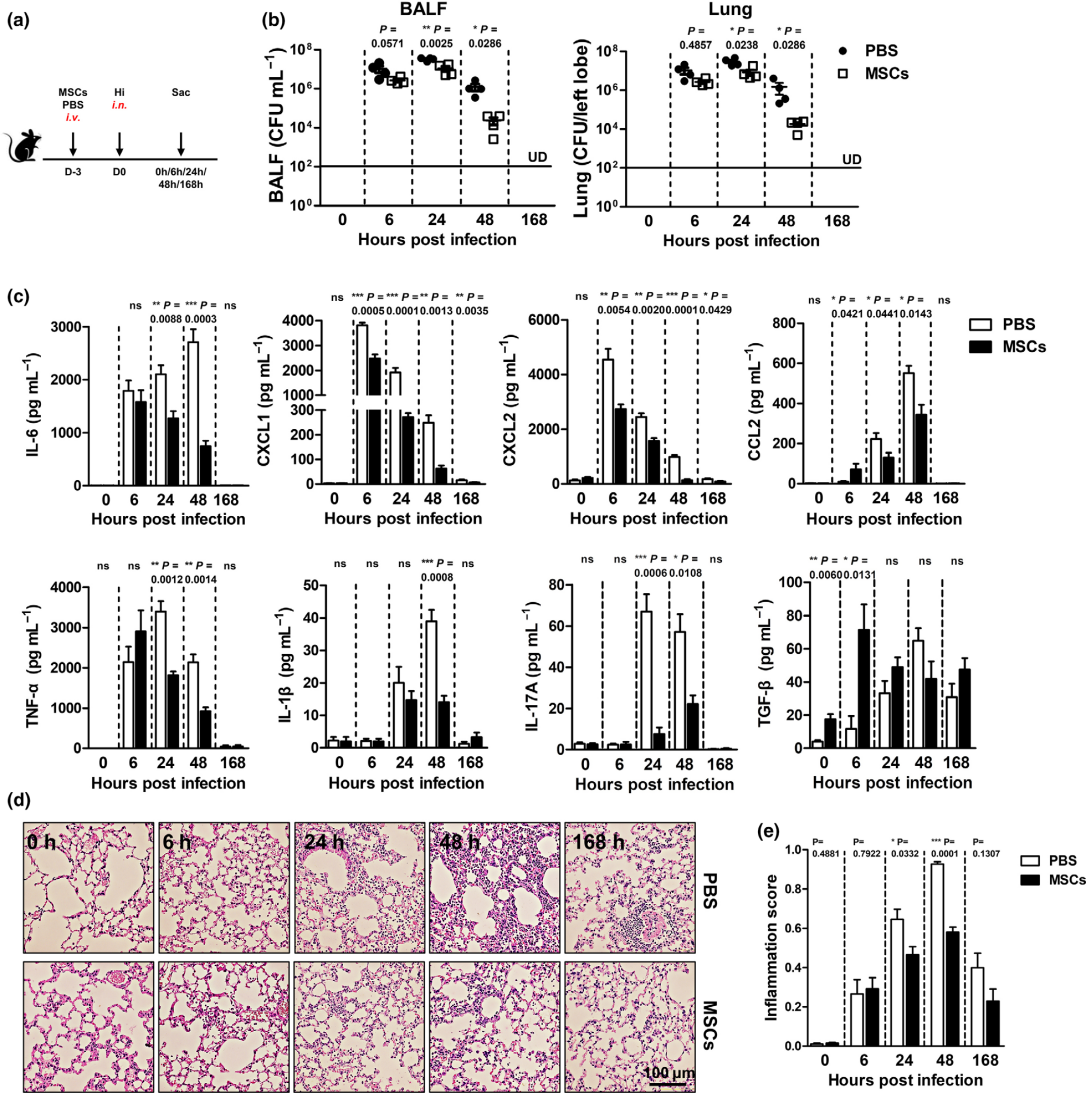
MSC‐preconditioned lung restricts infection‐induced inflammation and allows for better bacterial elimination. Three days after MSC infusion, mice were infected and sacrificed at the indicated time. **(a)** Scheme of the experiment. **(b)** Bacterial loads in the lung. **(c)** BALF cytokine levels. **(d)** Representative photographs of H&E staining. **(e)** Inflammation score. n = 4 each group; UD, undetected. ns, no significant difference. All the experiments were repeated three times, and representative data are shown.

To understand how MSC pretreatment reduced the severity of infection, we monitored the development of inflammatory responses in the lung and tried to figure out how they affected the elimination of bacteria (Figure 2a). At the early time point (6 h), PBS and MSC groups had similar numbers of bacteria in the lungs. Differences appeared at 24 h postinfection, when MSC group had threefold fewer bacteria than the control mice. The gap further widened into 50‐fold within the next 24 h. On day 7 (168 h), both groups eradicated the invading pathogens (Figure 2b). Then, we examined inflammatory responses at the corresponding time points. Pro‐inflammatory cytokines and chemokines play crucial roles in magnifying antimicrobial immunity by recruiting and activating immune cells, such as neutrophils and monocytes. Upon infection, we observed that less pro‐inflammatory cytokine was produced by the MSC‐preconditioned lungs. CXCL2 (MIP‐2) and CXCL1 (KC), which are responsible for the infiltration of neutrophils and monocytes, were suppressed at the beginning of infection (6 h), whereas inhibition of CCL2 (MCP‐1), TNF‐α, IL‐6, IL‐1β and IL‐17A appeared after 24 h. By day 7, although mice from control groups still had a few infiltrated cells surrounding the bronchi, inflammation was almost resolved in both the groups. Noteworthy, we found that secretion of TGF‐β (0 h and 6 h), which is involved in the anti‐inflammatory functions, significantly increased after MSC infusion (Figure 2c). Since overaccumulation of neutrophils and inflammatory monocytes leads to the destruction of pulmonary microvascular and alveolar epithelial cell barrier and subsequently impairs the bacterial clearance,^15^ we then evaluated the lung pathology to see whether MSC pretreatment reduced cell infiltration and prevented severe tissue damage. The results showed that infiltrations of immune cells were comparable between groups at the initial stage of Hi infection. However, as the inflammatory response developed, especially on day 2 after infection, control lungs displayed aggravated cell infiltration and serious tissue damage, while moderate interstitial thickening and less neutrophilic alveolar and interstitial infiltration were observed in the MSC‐preconditioned lungs (Figure 2d and e). Together, our data indicate that by preventing the overwhelming inflammation, MSC pretreatment well preserves the lung structures and strengthens the antibacterial immunity.

### Regulatory T cells increase dramatically in the MSC‐preconditioned lungs and are indispensable for protection

**Figure 3:**
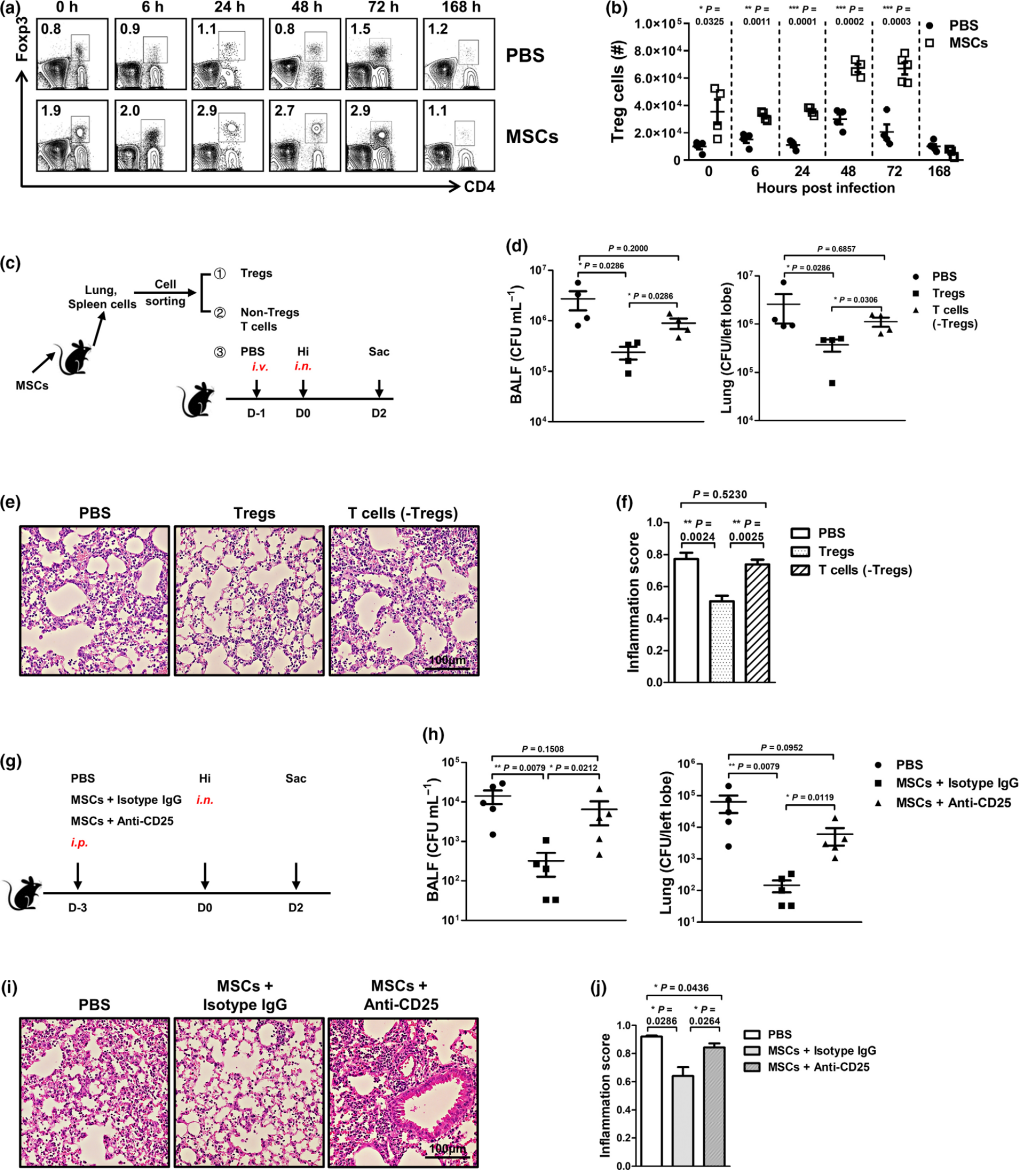
Regulatory T cells increase dramatically in the MSC‐preconditioned lungs and are indispensable for protection. After infusion of MSCs, mice were infected as described in Figure 2. To study the changes of Tregs in the lung, mice were sacrificed at the indicated time. **(a, b)** Representative FACS data and absolute number of Tregs. n = 4 or 5 per group. **(c)** Tregs and non‐Treg T cells were isolated from MSC‐treated mice and transferred separately to naïve mice. Twenty‐four hours later, mice were infected and sacrificed 2 days later. **(d–f)** Lung bacteria and pathology were determined. n = 4 per group. **(g)** To deplete Tregs, MSC‐treated mice were injected with anti‐CD25 antibodies intraperitoneally. Mice that receiving MSCs and isotype IgG or PBS alone were set as controls. Then, all the mice were infected. By day 2 after infection, bacterial loads and tissue inflammation in the lung were determined. **(h)** Bacteria numbers. **(i, j)** Lung pathology and scoring. n = 5 per group. All the experiments were repeated three times with similar results. ns, no significant difference. Representative data are shown.

Realising that MSC pretreatment benefited the recipient by preventing the overactivation of inflammatory responses, we sought to identify the immune cell population(s) that is responsible for the anti‐inflammatory functions. Regulatory T cells (Tregs) are required for controlling infection‐induced inflammation and promote tissue repair.^16^ In addition, several studies have described an increase in lung Tregs, which lasts more than 96 h after MSC therapy.^11^ Thus, we proposed that Tregs might be the anti‐inflammatory cells that are regulated by MSCs. To this end, we first determined and compared the numbers of Tregs in the two groups. The data showed that Tregs increased dramatically in the lung on day 3 after MSC treatment (0 h). The superiority was kept throughout the infection. Upon resolution of infection (168 h), the numbers of Tregs decreased and became similar in the two groups (Figure 3a and b). Notably, we found that only infusion of MSCs could induce dramatic increase in Tregs, while infusion of FLS did not significantly upregulate Tregs in the lung (Supplementary figure 2), implying that Tregs might be required for the protection. To verify this hypothesis, we isolated Tregs and non‐Treg T cells from the MSC‐treated mice and transferred them to the naïve mice separately, and then compared their ability to protect against the following infection (Figure 3c). As expected, Treg‐treated mice had about 10‐fold lower Hi load than those receiving PBS or non‐Treg T cells (Figure 3d). Moreover, they had moderate tissue damage and neutrophil infiltration (Figure 3e and f). On the contrary, depletion of Tregs with anti‐CD25 antibodies almost abrogated the protective effects of MSC pretreatment. Mice receiving both MSCs and anti‐CD25 antibodies exhibited similar antibacterial ability with the PBS‐treated ones (Figure 3g and h). Inhibition of infection‐induced inflammation by MSCs was also deprived by anti‐CD25 antibody treatment (Figure 3i and j). Thus, MSC‐induced Tregs are beneficial in facilitating appropriately robust and protective antimicrobial immune responses.

### MSC‐induced Tregs express chemokine receptor CXCR3 and exhibit enhanced anti‐inflammatory functions

**Figure 4:**
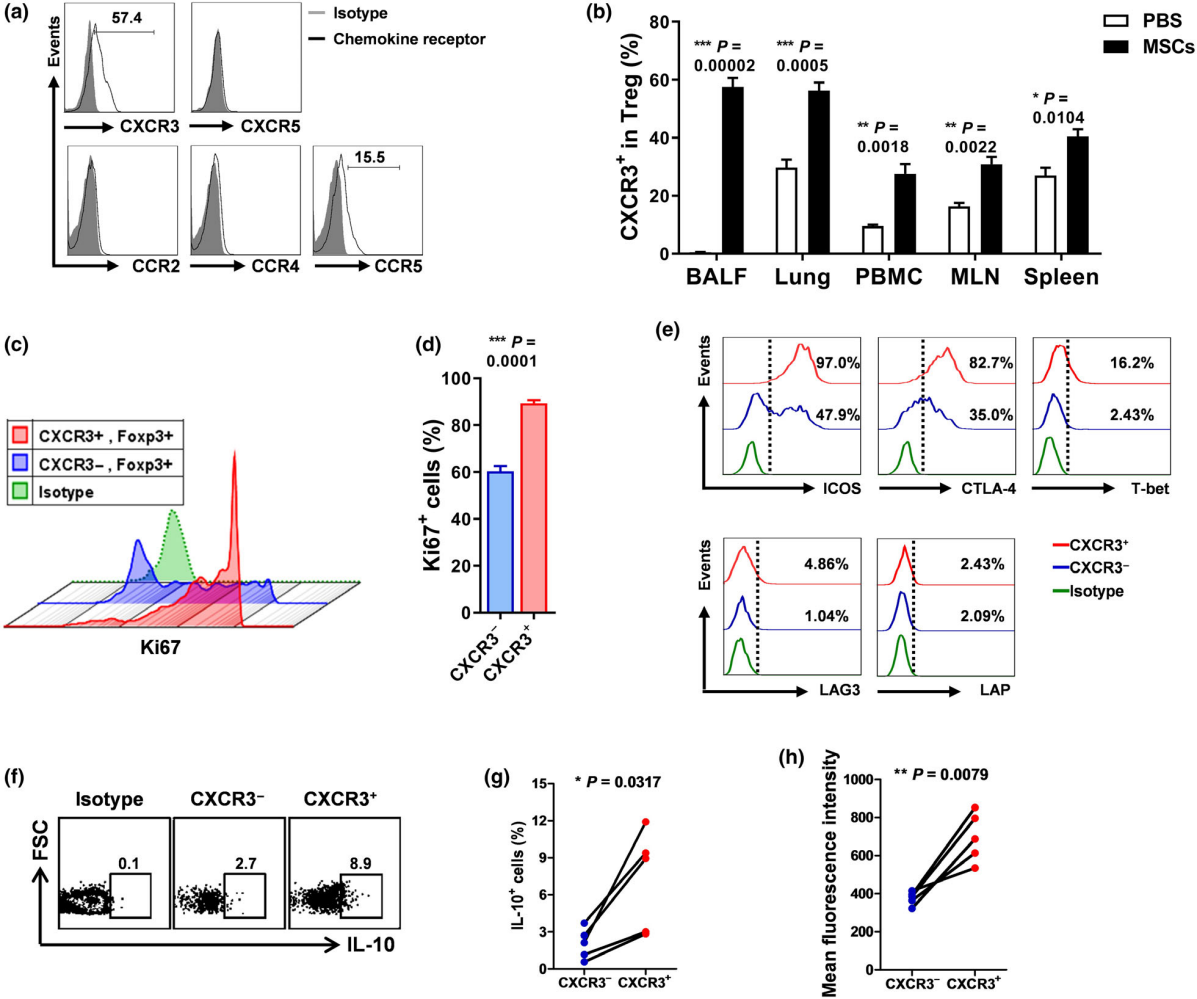
MSC‐induced Tregs highly express chemokine receptor CXCR3 and exhibit enhanced anti‐inflammatory functions. Mice were injected with MSCs or PBS. Three days later, mice were sacrificed. **(a)** Expression of chemokine receptors by lung Tregs was evaluated, and representative FACS graphs were shown. **(b)** Frequencies of CXCR3^+^ Tregs in lung and peripheral lymphoid tissues were determined. n = 3 per group. **(c, d)** Proliferative ability of CXCR3^−^ and CXCR3^+^ Tregs was compared. Expression of ki67 was measured. n = 5 per group. **(e)** Expression of ICOS, CTLA‐4, T‐bet, LAG3 and LAP was examined. Production of IL‐10 by CXCR3^−^ and CXCR3^+^ Tregs from MSC‐treated mice was determined by intracellular staining. **(f)** Representative FACS data. **(g)** Percentages of IL‐10‐producing Tregs. **(h)** Mean fluorescence intensity of IL‐10. n = 5 per group. All the experiments were repeated three times. Representative data are shown.

Previous studies showed that MSCs promoted the proliferation of Tregs by inducing TGF‐β secretion,^17^ which was also observed in our study (Figure 2c). However, the increase in lung Tregs could also be the result of recruitment from peripheral sites or a combination of the two. We next determined whether there were any Tregs recruited from peripheral sites. Migration of Tregs to the specific tissues depends on both the expression of chemokine receptors and the concentration gradient of the relevant chemokines in tissues. Therefore, we isolated lung Tregs from MSC‐treated mice and checked their expression of chemokine receptors. We found that about 60% of the Tregs highly expressed CXCR3 and only a small percentage of them (~15%) were positive for CCR5. CCR2, CCR4 and CXCR5 were barely detected (Figure 4a), implying that CXCR3 might drive the migration of Tregs to the lung. Then, we compared the percentages of CXCR3^+^ Tregs in the local (lung) and peripheral lymphoid tissues, including blood and the draining lymph node (mediastinal lymph node, MLN). CXCR3^+^ Tregs increased significantly in all the tissues after MSC infusion; however, lung had the highest percentage, indicating that CXCR3^+^ Tregs enriched there (Figure 4b). *In vivo* CD45 staining is able to discriminate resident cells from circulating cells successfully. We injected anti‐CD45 antibodies to the mice intravenously and stained the circulating Tregs. The data showed that the percentage of CXCR3^+^ Tregs in the lung capillary (40%) was comparable to that in the lung tissue (50%) and was much higher than that in the peripheral blood (25%), further evidencing the migration of CXCR3^+^ Tregs to the lung (Supplementary figure 3).

Tregs are a heterogeneous population with respect to their functional activity and activation status. They will specialise their anti‐inflammatory functions according to the encountering environment. Thus, to understand how CXCR3^+^ Tregs protect against infection, we characterised the functions of them and found that more than 90% of them expressed ki67, a marker of proliferating cells, while only about 60% of CXCR3^−^ Tregs were positive for ki67 (Figure 4c and d). T‐bet, which has been reported to control the development of CXCR3^+^ Tregs, was upregulated in the CXCR3^+^ Tregs as expected. Expression of co‐inhibitory receptor CTLA‐4 and LAG‐3 was much higher in CXCR3^+^ Tregs. Expressing latency‐associated peptide (LAP) by Tregs indicates that they function in a TGF‐β‐dependent manner^18^; however, LAP was barely detected in the surfaces of CXCR3^+^ Tregs (Figure 4e), suggesting that TGF‐β might not be the main anti‐inflammatory molecule. ICOS, which enables T cells preferentially to produce IL‐10^19^, ^20^, was detected almost in all the CXCR3^+^ Tregs (Figure 4e). Indeed, we found that CXCR3^+^ Tregs secreted more IL‐10 than CXCR3^−^ ones (Figure 4f–h). Together, we show that Tregs induced by MSC treatment express chemokine receptor CXCR3, and obtain stronger immunosuppressive functions.

### MSC treatment induces production of CXCL9 and CXCL10 by lung phagocytes and recruits CXCR3^+^ Tregs

**Figure 5:**
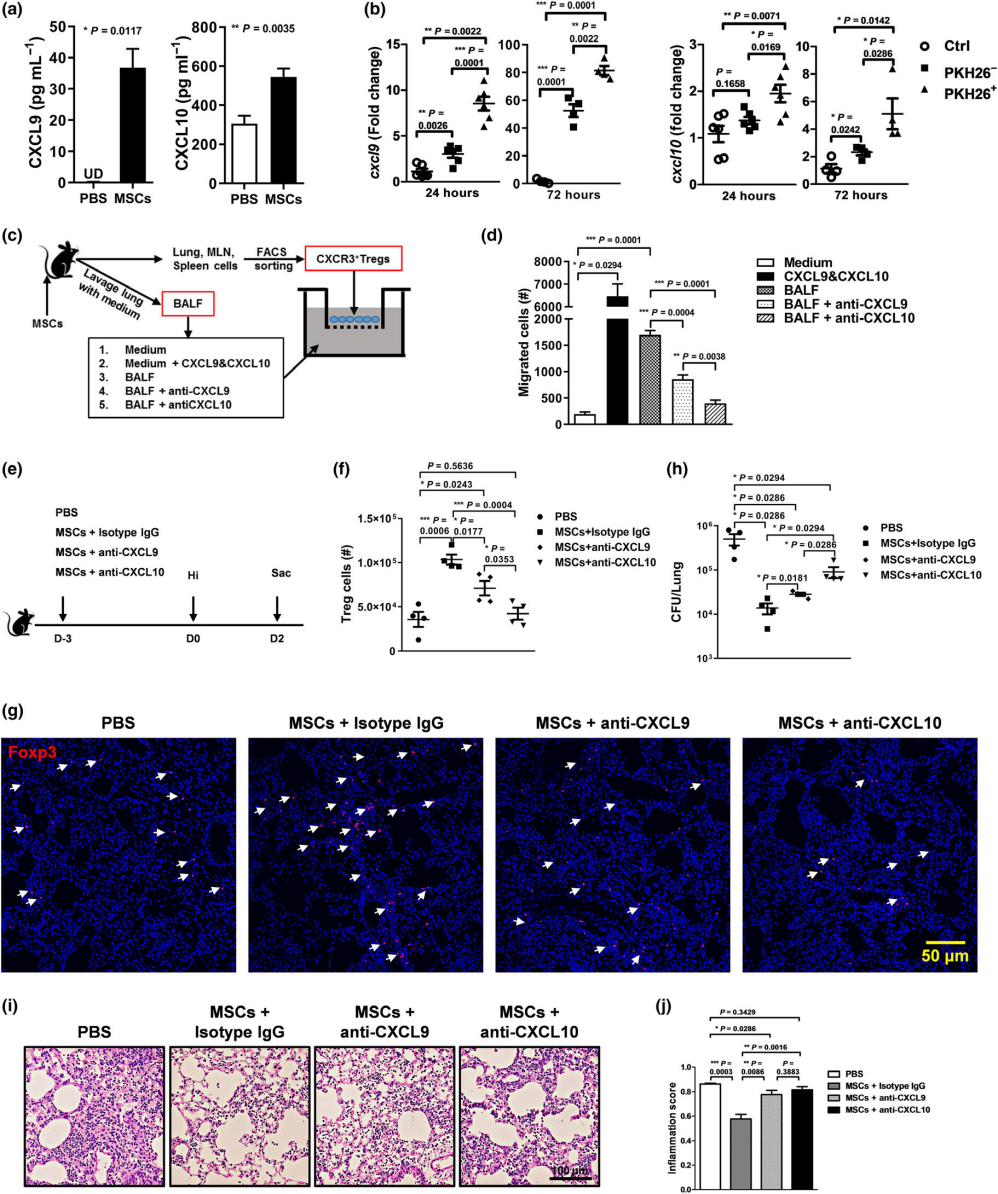
MSC treatment induces production of CXCL9 and CXCL10 by lung phagocytes and recruits CXCR3^+^ Tregs. Lung lavage was carried out 3 days after MSC infusion. **(a)** Concentrations of CXCL9 and CXCL10 in the BALF were measured by ELISA. n = 5 per group. **(b)** PBS or PKH26‐labelled MSCs were injected into mice intravenously. After 24 and 72 hours, lungs cells were isolated and phagocytes that had engulfed MSCs (PKH28^+^) or not (PKH26^−^) were sorted by FACS, respectively. Their expression of *cxcl9* and *cxcl10* was determined by RT‐PCR. Phagocytes isolated from PBS‐treated lung were set as controls. n = 5 or 6 per group. **(c, d)** CXCR3^+^ T cells were collected from MSC‐treated mice. Chemotaxis of CXCR3^+^ T cells to CXCL9 and CXCL10 was assayed. n = 4 per group. **(e)** Two hours after MSC infusion, mice receiving neutralisation antibodies anti‐CXCL9 or anti‐CXCL10 or isotype antibodies via the intraperitoneal route, and then, they were infected and sacrificed 48 hours later. PBS‐treated mice were used as controls. Tregs in the lung were determined by FACS **(f)** and histological immunofluorescence **(g)**. Foxp3 (red) and nuclei (blue). Arrowhead = Treg. **(h)** Lung bacterial loads. **(i, j)** Lung pathology. Representative photographs and inflammation scores were shown. n = 4 each group; UD, undetected. All the experiments were repeated three times. Representative data are shown.

CXCL9, CXCL10 and CXCL11 are the ligands of CXCR3.^21^, ^22^ Since C57BL/6 mice are natural null mutants for *cxcl11*, we then measured the production of CXCL9 and CXCL10 in the lungs. Both of the two chemokines increased markedly in the BALF collected from MSC‐treated mice (Figure 5a). Macrophages, monocytes and dendritic cells are the main source of CXCL9 and CXCL10.^23^ Our study and others showed that MSCs were engulfed by phagocytic cells, more than half of which expressed the classical macrophage marker F4/80 (Supplementary figure 4a). These clues suggested that after engulfing the injected MSCs, phagocytes might upregulate the expression of CXCL9 and CXCL10. To confirm the hypothesis, we isolated phagocytes that had engulfed MSCs from those who had not and compared their production of CXCL9 and CXCL10. MSCs were stained with PKH26, a cell‐membrane‐binding fluorescent dye, and transferred into mice. CD45^+^F4/80^+^PKH26^+^ and CD45^+^F4/80^+^PKH26^−^ cells were sorted 24 and 72 h later, respectively (Supplementary figure 4b). CD45^+^F4/80^+^ cells isolated from PBS‐treated lung were used as controls. Expression of *cxcl9* and *cxcl10* by them was then determined. Although *cxcl9* and *cxcl10* increased in both PKH26^−^ and PKH26^+^ phagocytes, their expression was much higher in the later subset. As the time extended, the differences between the two subsets contracted gradually (Figure 5b). Thus, phagocytosis of MSCs triggers the production of CXCL9 and CXCL10.

To confirm that recruitment of CXCR3^+^ CD4 T cells is mediated by CXCL9 and CXCL10, we isolated the cells and determined their migration to the two chemokines via the Transwell system. Medium alone was set as the negative control, while recombinant CXCL9 and CXCL10 proteins were used as the positive control. We saw that both recombinant CXCL9 and CXCL10 proteins and BALF collected from MSC‐treated mice could chemoattract CXCR3^+^ CD4 T cells. Neutralisation of CXCL9 or CXCL10 in BALF collected from MSC‐treated mice significantly blocked the migration of CXCR3^+^ CD4 T cells (Figure 5c and d). What's more, neutralisation of CXCL9 or CXCL10 *in vivo*, especially the later one, dramatically inhibited the MSC‐induced migration of Tregs to the lung (Figure 5e–g) and reduced the protective effects of MSCs (Figure 5h–j). Together, phagocytosis of MSCs by lung phagocytes promotes the production of CXCL9 and CXCL10, which then leads to the recruitment of CXCR3^+^ Tregs.

### MSC pretreatment protects lupus‐prone mice from pulmonary infection

**Figure 6:**
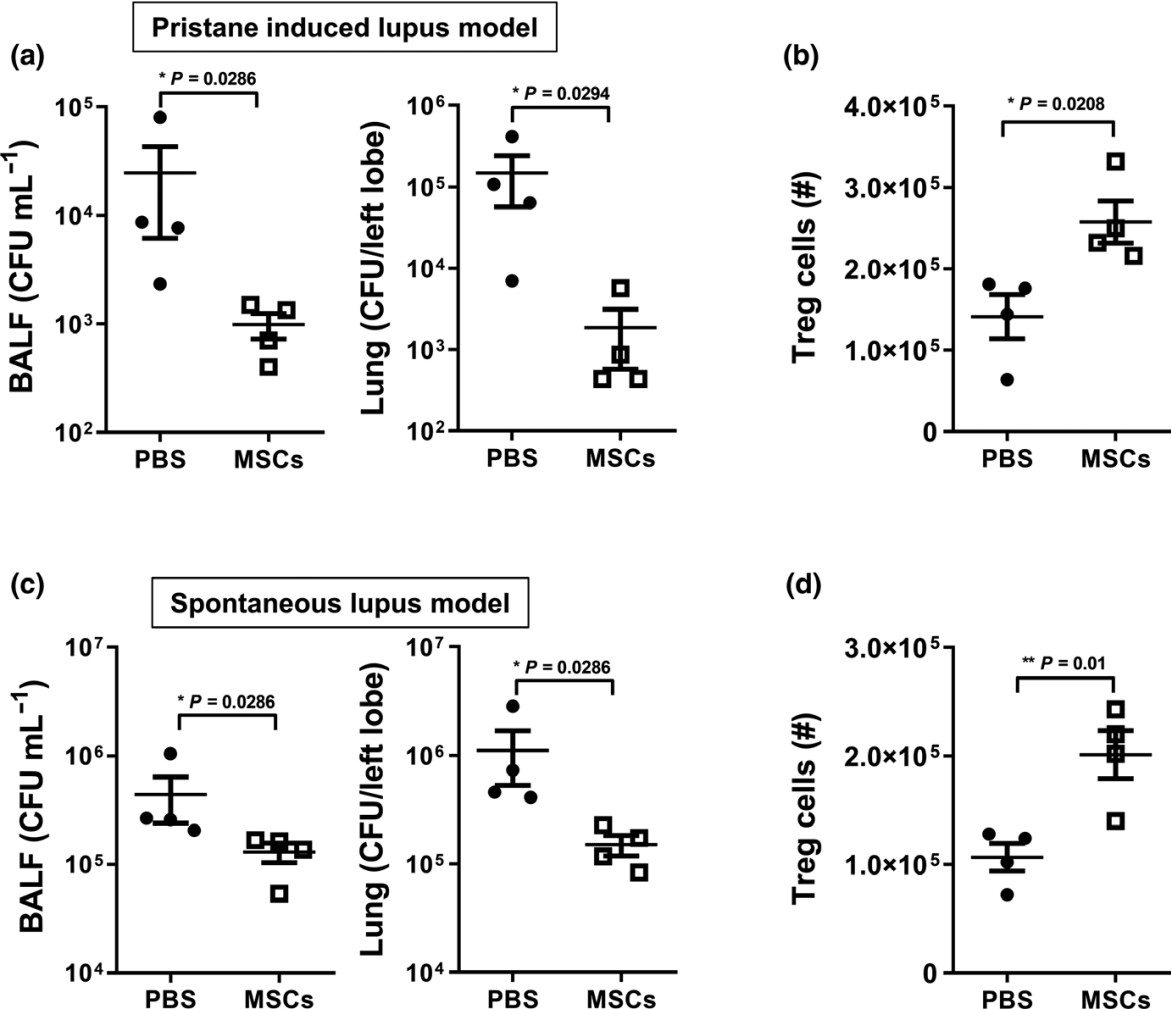
MSC pretreatment protects lupus‐prone mice from pulmonary infection. Pristane‐induced lupus mice and B6. *lpr* mice were treated with PBS or MSCs and then were infected by Hi as described in Figure 1c. On day 2 after infection, bacteria and Tregs in the lungs of pristane‐induced lupus mice **(a, b)** and B6. *lpr* mice **(c, d)** were determined. n = 4 per group. All the experiments were repeated three times. Representative data are shown.

Considering that the immune system changes a lot after the autoimmunity developed, we cannot arbitrarily assume that MSC treatment protects autoimmune mice as well. The improved antimicrobial immunity seen in the SLE patients who received MSCs could result from suppression of autoimmune responses. Thus, we next employed the lupus‐prone mice to assess the protective effects of MSCs in the setting of autoimmunity. Because none of the commonly used lupus‐prone murine strains perfectly mimics human SLE disease, we decided to determine the protective effects of MSCs in both the induced (pristane‐induced) (Figure 6a and b) and spontaneous (B6.*lpr*, defective in Fas‐mediated signalling) lupus models (Figure 6c and d). Bacterial counting data showed that clearance of invading Hi by both the induced and spontaneous lupus mice were sped up by MSC treatment (Figure 6a and c). Meanwhile, after infusing MSCs, the numbers of lung Tregs increased remarkably in both of the lupus mice (Figure 6b and d). Thus, our study shows that MSC therapy could reduce the severity of the following respiratory bacterial infection by increasing Tregs, which subsequently prevent overactivated inflammation in the lupus mice.

## Discussion

The most unwanted side effect of the current immunosuppressants is increasing the susceptibility of patients to infection.^24^ Thus, avoiding the detrimental effects of these drugs on the antimicrobial immunity or developing efficient preventive strategy is urgently needed. MSC therapy has been shown to not only alleviate the autoimmune disease symptoms, but also reduce the incidence and/or duration of respiratory tract infection.^8^ This observation is interesting and inspiring. However, what is the underlying mechanism? Does MSC therapy directly prevent infection? Or it is just the beneficial result of the control of autoimmunity. In this study, to exclude the interference of the latter situation, we first employed the wild‐type mice to evaluate the preventive effects of MSC therapy. After intravenous infusion, MSCs were engulfed by lung phagocytes, which then secreted CXCL9 and CXCL10 and recruited tremendous CXCR3^+^ Tregs. During the following infection, these Tregs strongly inhibited the tissue‐damaging inflammation and allowed for better bacterial clearance. More importantly, MSCs protected mice with autoimmune disease from severe infection via the same manner.

MSCs have been employed to treat acute infection for a long time, including sepsis and acute lung injury caused by *Escherichia coli*, *Pseudomonas aeruginosa* and *Klebsiella pneumoniae*.^25^, ^26^, ^27^, ^28^. In these studies, MSCs were able to recognise microorganisms and kill them by secreting antimicrobial peptides. Besides, stimulated by inflammatory cytokines TNF‐α and IFN‐γ, MSCs could increase IDO production and exhibited a broad‐spectrum antimicrobial function.^13^ Thus, one may assume that MSCs prevent infection by directly killing the invading bacteria. However, several studies also reported that intravenously transfused MSCs accumulated in the lung and transmigrated outside of the vascular space, where more than half of them were rapidly phagocytosed by lung‐resident macrophages and disappeared within 24 h.^12^ Here, we observed a similar phenomenon; thus, the long‐term preventative effects cannot be attributed to the antimicrobial properties of MSCs.

Tregs play an indispensable role in resolving infection by orchestrating the magnitude of effector responses and limiting collateral tissue damage caused by vigorous immune responses.^29^ Deficiency in Treg functions led to the occurrence of hyperinflammation and subsequently facilitated bacterial growth.^30^, ^31^ In treating LPS‐induced acute lung injury and caecal ligation and puncture (CLP) sepsis, MSCs ameliorated disease symptoms by inducing Tregs to balance the anti‐ and pro‐inflammatory factors.^32^, ^33^ In our model, where MSCs were transferred to the sterile animals, they still induced a systemic increase in Tregs, especially in the lung. Interestingly, these pre‐existing Tregs benefited the host by keeping a delicate balance of allowing for antipathogenic immunity and preventing immune pathology. Different from bacterial infection, the roles of Tregs in the fungal and viral are quite ambiguous. Early studies on *Leishmania major*, HSV‐1 and other infection suggested that immune response developed in the absence of Tregs was better able to target the pathogen, indicating that Tregs helped the pathogens to escape from the host immunity.^34^, ^35^, ^36^ However, in mucosal HSV‐2 infection, Soerens *et al*.^37^ reported that Tregs were essential to promote proper CD4 T‐cell priming and required for controlling viral replication. Pandemic of coronavirus disease 2019 (COVID‐19), caused by severe acute respiratory syndrome coronavirus 2 (SARS‐CoV‐2), has taken lives of more than 62,000 people globally (as of July 23, 2020). Growing information suggested that the persistence of virus‐induced cytokine storm drove severe pathogenesis and led to death.^38^ Considering their powerful immunomodulatory ability and success in alleviation of acute lung injury, MSC‐based therapies have been initiated in China for COVID‐19 respiratory disease.^39^ Preliminary clinical data showed that pneumonia was greatly relieved by MSC transplantation.^40^ Thus, MSCs played a positive role in combating with SARS‐CoV‐2. Since this virus is highly contagious, vaccine or other preventive strategy is the ideal choice to control the disease. Unfortunately, so far no vaccine is available. In our study, we pointed out that MSCs could induce Tregs to prevent severe inflammation induced by infection. Therefore, it would be interesting and attractive to see whether transplantation of MSCs to the high‐risk group, like the elders in advance, might be able to reduce the incidence of severe cases.

The mechanisms of MSC‐induced increase in Tregs have been extensively investigated.^41^ A study by Akiyama *et al*.^17^ showed that intravenous transfer of mouse bone marrow MSCs to naïve mice induced T‐cell apoptosis in peripheral blood and bone marrow. The apoptotic T cells stimulated macrophages in the spleen to produce TGF‐β, which subsequently promoted the proliferation of Tregs. In another study, Braza *et al*.^42^ found that, after infusion of MSCs, circulating monocytes displayed an immunosuppressive phenotype and secreted IL‐10 or TGF‐β to induce the differentiation of Tregs in the peripheral blood. However, how MSCs promote the increase of Tregs in the local tissues, for example the lung, needs to be elucidated. Both proliferation of resident Tregs and recruitment of the peripheral Tregs could account for the increase. Here, we detected a slight increase of TGF‐β in the lung after MSC infusion, but the quantity was not as abundant as the aforementioned study showed. Thus, we turned to check the expression of chemokine receptors by the MSC‐induced Tregs. The results showed that the majority of BALF Tregs (>60%) exclusively expressed high level of CXCR3. Besides, phagocytes that engulfed MSCs secreted large amount of CXCR3 ligands, CXCL9 and CXCL10. Employing neutralising antibodies, we confirmed their role in recruiting CXCR3^+^ Tregs. Thus, our study provided a novel mechanism of how MSCs increased the number of Tregs in the local tissues. In response to different environmental stimuli, Tregs could co‐express Foxp3 with other Th cell lineage‐specific transcription factors, which sequentially initiate expression of related chemokine receptors such as T‐bet/CXCR3 (Th1), Stat3/CCR6 (Th17) and IRF4/CCR4 and further specialise their anti‐inflammatory functions to the corresponding effector responses.^43^ Therefore, it is interesting and important for continuing studies to explain how MSC treatment instructs Tregs to upregulate CXCR3, which indubitably will lead to deeper understanding of immunoregulatory properties of MSCs.

Tregs restrict infection‐induced inflammation via multiple mechanisms, including contact‐dependent inhibitory cell surface receptors (CTLA‐4 and LAG‐3), secretion of inhibitory cytokines (IL‐10 and TGF‐β) and direct lysis (via granzymes).^16^, ^43^ By examining the functionary molecules of CXCR3^+^ Tregs, higher levels of CTLA‐4 and LAG‐3 were detected. Notably, nearly all of them were positive for ICOS. ICOS is a CD28 superfamily‐related molecule, which plays an important role in T‐cell activation/survival. Employing the murine diabetes model, Kornete *et al*.^19^, ^20^ found that ICOS^+^ Treg subset, in contrast to its ICOS^−^ counterpart, was endowed with the prominent proliferative and suppressive capacities *in situ* and readily secreted IL‐10 upon islet‐Ag stimulation. Ina continuous study, they showed that ICOS^+^ Tregs preferentially homed to the inflamed islets using CXCR3. In line with their discovery, we find that CXCR3^+^ Tregs produce more IL‐10 than CXCR3^−^ Tregs, implying that CXCR3^+^ Tregs may secrete IL‐10 to modulate the infection‐induced inflammation.

Increased infection risk is the inevitable side effect of long‐term usage of immunosuppressive drugs. No effective countermeasure is available. Recently, MSCs have become a novel therapeutic choice for autoimmune diseases and other immune disorders. By finding that MSCs are able to reduce the severity of following respiratory infection, our study makes them a more attractive choice for clinicians to manage the diseases that need a long‐term immunosuppressant regimen.

## Methods

### Animals

Female 7‐ to 8‐week‐old C57BL/6 and 14‐week‐old B6.*lpr* mice were purchased from Nanjing Medical University Animal Core (Nanjing). Mice were housed in the animal facility at Nanjing Drum Tower Hospital. All the experimental protocols were approved by the Ethics Committee for Animal Research in the Affiliated Drum Tower Hospital of Nanjing University Medical School (ref: 2016‐206‐01).

### Pristane‐induced lupus model

Female 7‐ to 8‐week‐old C57BL/6 mice received a single intraperitoneal injection of 0.5 mL of pristane (Sigma‐Aldrich, Missouri, United States) and developed lupus symptoms after 6 months.^44^

### Preparation of MSCs

MSCs were isolated as described in our previous study. Briefly, umbilical cords from scheduled healthy caesarean sections were collected. Then, they were cut into 1‐cm‐long pieces and finely minced and digested with CDH buffer (250 U mL^−^^1^ collagenase II, 100 U mL^−^^1^ dispase and 100 U mL^−^^1^ hyaluronidase in DMEM/F12 medium) at 37°C on an orbital shaker for 4 h. The suspension was then diluted 1:5 with PBS at room temperature and centrifuged at 840 g for 10 min. Cells were plated at 4000–6000 cells per cubic centimetre in the media, and nonadherent cells and debris were removed after 48 h. Adherent cells were cultured, and at passages 6–8, cells were collected and used in all the experiments. The phenotype of the resulting MSCs was determined by surface staining as described in our previous study.^45^ Human synovial fibroblasts (FLS) were purchased from Otwo Biotech (ShenZhen, China) Inc. (Catalogue No. HTX2177) and cultured following the instructions.

### Lung *H. influenzae* infection model

Lung infection was set up as previously reported.^46^ Briefly, mice were anaesthetised by intraperitoneal (*i.p*.) injection with ketamine/xylazine and inoculated with ~1 × 10^8^ CFU of Hi in 30 μL PBS intranasally. To enumerate bacteria, lungs were homogenised in 1 mL sterile PBS, and the resulting homogenates were subjected to serial 10‐fold dilution. A total of 10 μL of each dilution was plated on agar plates supplemented with nicotinamide adenine dinucleotide (5 μg mL^−^^1^) and hemin (10 μg mL^−^^1^) (sBHI) (Sigma‐Aldrich). Bacterial colonies were counted after overnight culture at 37°C.

### Bronchial alveolar lavage and lung digestion

Bronchial alveolar lavage (BAL) was obtained by cannulating the trachea with a 20‐gauge catheter. Lung was lavaged with cold PBS. BALF was centrifuged, and the cell‐free supernatants were stored at −80°C. Lungs were minced and incubated at 37°C in Isocove's DMEM containing collagenase A and DNase I (Roche, Switzerland). The resulting cell suspension was then mashed through a 40‐μm cell strainer.^46^

### Enzyme‐linked immunosorbent assay

For the quantitative determination of BALF cytokine protein levels, ELISA kits for murine IL‐1β, IL‐6, IL‐17, TNF‐α, CXCL1 (KC), CXCL2 (MIP‐2), CCL2 (MCP‐1), TGF‐β, CXCL9 and CXCL10 were used (R&D Systems, Minnesota, United States; BioLegend, California, United States). All the assays were performed according to the manufacturer's instructions.

### Lung pathology

Lungs were perfused from the right ventricle of the heart with 10 mL isotonic saline, then removed and fixed. Four‐micrometre sections were cut and stained with haematoxylin and eosin (H&E). Following the reported grading guidelines, a pathologist blindly scored each lung. Ten fields per section from each sample were determined.^47^

### Flow cytometry and intracellular cytokine staining

Lung cells were stained after FcγRII/III blocking with anti‐mouse CD16/CD32 (clone 93; eBioscience, California, United States). The following anti‐mouse mAbs purchased from Becton, Dickinson and Company (BD, New Jersey, United States), BioLegend or eBioscience were used: CD45 (30‐F11), CD11c (N418), F4/80 (BM8), CD4 (RM4‐5), CD25 (PC61.5), CXCR3 (CXCR3‐173), CXCR5 (SPRCL5), CCR2 (SA203G11), CCR4 (2G12), CCR5(HM‐CCR5), Foxp3 (FJK‐16s), T‐bet (eBio4B10), Ki67 (SolA15), LAG3 (C9B7W), ICOS (15F9), CTLA (UC10‐4B9), LAP (TW7‐16B4) and IL‐10 (JES5‐16E3). For the intracellular staining of Foxp3, T‐bet, Ki67 and IL‐10, cells were surface‐stained with CD4 and followed by permeabilisation with Foxp3 staining kit (eBiosciences). Then, the cells were stained with Foxp3, Ki67, IL‐10 and T‐bet. FACSCalibur or FACS Fortessa (BD) were employed to collect the data. Data were then analysed with FlowJo software.

### Adoptive transfer experiments

Three days after MSC infusion, mice were sacrificed. T cells of lung and spleen were purified by using Pan T Cell Isolation Kit (Miltenyi Biotec, Germany), and purity of the sorted cells was determined by FACS (Supplementary figure 5a). CD4^+^CD25^+^ cells were then sorted out of the purified T cells by FACSAria, and the purity was determined by FACS (Supplementary figure 5b). Non‐Treg T cells were also collected. Approximately 1 × 10^6^ cells were adoptively transferred into naïve recipients via the intravenous route.

### *In vivo* depletion of Treg cells

After receiving MSCs intravenously, mice were immediately given *i.p*. injections of 0.3 mg of anti‐CD25 (clone PC61) or control rat IgG antibodies purchased from Bio X Cell (New Hampshire, United States) as described everywhere.^17^ On day 3 post‐antibody treatment, mice were infected with Hi. Two days after infection, bacteria in the lung homogenates were enumerated. Depletion of CD4^+^CD25^+^ cells was confirmed by flow cytometry (Supplementary figure 5c).

### Isolation of lung phagocytes by cell sorting

MSCs were labelled with PKH26 dye, according to the manufacturer's protocol (Sigma). Briefly, cell pellets were resuspended in 1 mL diluent C. Separately, 1 mL diluent C was mixed with 4 μL PKH26. The cell suspension was mixed with the stain solution and incubated at room temperature. The labelling reaction was stopped by adding an equal volume of FBS. Labelled cells were centrifuged and washed twice with PBS. One or three days after infusion of PKH26‐stained MSCs, total lung cells were obtained. Then, they were labelled with Fixable Viability Stain 700 (FVS700) (BD), anti‐CD45‐PerCP‐Cy5.5 and anti‐F4/80‐BV421. The live cells were sorted after gating for viability (negative for FVS700), morphology (FSC‐H vs. FSC‐A), expression of CD45, F4/80 and PKH26 dye.

### Real‐time PCR

Total RNA from lungs or sorted cells was extracted by using Total RNA Extraction Reagent, and complementary DNA was prepared by using HiScript II 1st Strand cDNA Synthesis Kit (Vazyme Biotech, China). Quantitative PCR was performed with AceQ qPCR SYBR Green Master Mix (High ROX Premixed, Vazyme). Primer sequences for gene expression were as follows: CXCL9, 5′‐TAGGCAGGTTTGATCTCCGT‐3′ and 5′‐CGATCCACTACAAATCCCTCA‐3′; CXCL10, 5′‐CCTATGGCCCTCATTCTCAC‐3′ and 5′‐CTCATCCTGCTGGGTCTGAG‐3′; and GAPDH, 5′‐ATGGGGAAGGTGAAGGTCG‐3′ and 5′‐GGGGTCATTGATGGCAACAATA‐3′. The expression of each target gene relative to GAPDH was determined according to the ΔΔC_t_ method.

### Chemotaxis assay of CXCR3^+^ Tregs

To determine whether CXCL9 and CXCL10 recruited CXCR3^+^ Tregs, chemotaxis assays were performed. MSC‐treated lungs were lavaged with 2 mL of RPMI 1640 medium supplemented with 10% FBS and penicillin and streptomycin. Then, the resulting BALF was centrifuged and the supernatant was collected and combined. CXCR3^+^ CD4 T‐cell isolation: lungs, mediastinal lymph nodes and spleens were collected from MSC‐treated mice. Single‐cell suspensions were prepared, and T cells were purified by antibody‐coupled magnetic beads. Then, CXCR3^+^ CD4 T cells were sorted out of the purified T cells by a FACSAria. Chemotaxis assay: medium alone and medium containing 200 ng mL^−^^1^ of CXCL9 and CXCL10 (Proteintech, Illinois, United States) were used as negative and positive controls, respectively. 200 ng mL^−^^1^ of anti‐CXCL9 or 1 μg mL^−^^1^ anti‐CXCL10 antibodies (R&D) was added into the lower Transwell chamber (96‐well, Corning, New York, United States) that containing 150 μL of BALF. Then, about 1 × 10^4^ of CXCR3^+^ CD4 T cells were added into the upper Transwell chamber in a total volume of 100 μL. Plates were incubated at 37°C in 5% CO_2_ for 2 h. The contents of the lower well were then collected, and cells were counted in a haemacytometer. Cell migration to medium alone was used as control. All assays were performed in triplicate.

### Neutralisation of CXCL9 and CXCL10

For CXCL9 and CXCL10 blockade, mice were treated with MSCs intravenously. Two hours later, they were injected with 200 μg of either CXCL9 or CXCL10 (R&D) neutralising antibodies via the intraperitoneal route.^48^ Three days later, the mice were infected by Hi.

### Confocal microscopy and indirect immunofluorescence staining of Foxp3

Paraffin sections of lungs were deparaffinised, rehydrated and then boiled in the citrate buffer to retrieve antigens. Next, the resulted sections were permeabilised, blocked and incubated with the rabbit anti‐mouse Foxp3 antibodies (clone D6O8R, Cell Signaling Technology, Massachusetts, United States) at 4°C overnight. Then, the sections were incubated with secondary antibodies (Alexa Fluor 647‐conjugated goat anti‐rabbit IgG, FMS‐Rbaf64701) (Fcmacs Biotech, China) for 1 h at room temperature. Sections were kept in a dark environment. Nuclei were stained with 4′,6‐diamidino‐2‐phenylindole (DAPI) (KeyGen Biotech, China). The colocalisation of Foxp3 with nuclei was checked by confocal laser scanning microscopy (FV3000 Microscope, Olympus, Japan). Images were captured and processed by the software provided by the manufacturer.

### Statistical analysis

All analyses were performed by using Prism software (GraphPad 8.0). Data were presented as means ± SEM. The two‐tailed Student's *t*‐test was used for comparisons between two groups. The Kruskal–Wallis test was used to evaluate variance among all groups. If a significant variance was found, the Mann–Whitney *U*‐test was used to determine significant differences between individual groups. *P* < 0.05 was considered to be statistically significant. *P*‐values are depicted as follows: *P < 0.05; **P < 0.01; ***P < 0.001.

## Conflict of Interest

The authors declare no conflict of interest.

## Author Contribution

**Wenchao Li:** Data curation; formal analysis; investigation; methodology; project administration; writing – original draft; writing – review and editing. **Weiwei Chen:** Investigation; methodology; writing – review and editing. **Saisai Huang:** Methodology. **Genhong Yao:** writing – review and editing. **Xiaojun Tang:** Investigation; methodology; supervision. **Lingyun Sun:** Project administration; writing – review and editing.

## Supporting information

¶ Click here for additional data file.

## References

[1] Naji A , Eitoku M , Favier B , Deschaseaux F , Rouas‐Freiss N , Suganuma N . Biological functions of mesenchymal stem cells and clinical implications. Cell Mol Life Sci 2019; 76: 3323–3348. 31055643 10.1007/s00018-019-03125-1 PMC11105258

[2] Shi Y , Hu G , Su J *et al* Mesenchymal stem cells: a new strategy for immunosuppression and tissue repair. Cell Res 2010; 20: 510–518. 20368733 10.1038/cr.2010.44

[3] Martin I , Galipeau J , Kessler C , Le Blanc K , Dazzi F . Challenges for mesenchymal stromal cell therapies. Sci Transl Med 2019; 11: eaat2189. 30787168 10.1126/scitranslmed.aat2189

[4] Galipeau J , Sensebe L . Mesenchymal stromal cells: clinical challenges and therapeutic opportunities. Cell Stem Cell 2018; 22: 824–833. 29859173 10.1016/j.stem.2018.05.004 PMC6434696

[5] Meyer‐Olson D , Witte T . Immunology: prevention of infections in patients with autoimmune diseases. Nat Rev Rheumatol 2011; 7: 198–200. 21304507 10.1038/nrrheum.2011.14

[6] Siripaitoon B , Lertwises S , Uea‐Areewongsa P , Khwannimit B . A study of Thai patients with systemic lupus erythematosus in the medical intensive care unit: epidemiology and predictors of mortality. Lupus 2015; 24: 98–106. 25149601 10.1177/0961203314548884

[7] Cervera R , Khamashta MA , Font J *et al* Morbidity and mortality in systemic lupus erythematosus during a 10‐year period: a comparison of early and late manifestations in a cohort of 1,000 patients. Medicine (Baltimore) 2003; 82: 299–308. 14530779 10.1097/01.md.0000091181.93122.55

[8] Liang J , Zhang H , Kong W *et al* Safety analysis in patients with autoimmune disease receiving allogeneic mesenchymal stem cells infusion: a long‐term retrospective study. Stem Cell Res Ther 2018; 9: 312. 30428931 10.1186/s13287-018-1053-4 PMC6236873

[9] Agrawal A , Murphy TF . Haemophilus influenzae infections in the H. influenzae type b conjugate vaccine era. J Clin Microbiol 2011; 49: 3728–3732. 21900515 10.1128/JCM.05476-11 PMC3209133

[10] Goldblatt F , Chambers S , Rahman A , Isenberg DA . Serious infections in British patients with systemic lupus erythematosus: hospitalisations and mortality. Lupus 2009; 18: 682–689. 19502263 10.1177/0961203308101019

[11] Ko JH , Lee HJ , Jeong HJ *et al* Mesenchymal stem/stromal cells precondition lung monocytes/macrophages to produce tolerance against allo‐ and autoimmunity in the eye. Proc Natl Acad Sci USA 2016; 113: 158–163. 26699483 10.1073/pnas.1522905113 PMC4711840

[12] de Witte SFH , Luk F , Sierra Parraga JM *et al* Immunomodulation by therapeutic mesenchymal stromal cells (MSC) is triggered through phagocytosis of MSC by monocytic cells. Stem Cells 2018; 36: 602–615. 29341339 10.1002/stem.2779

[13] Meisel R , Brockers S , Heseler K *et al* Human but not murine multipotent mesenchymal stromal cells exhibit broad‐spectrum antimicrobial effector function mediated by indoleamine 2,3‐dioxygenase. Leukemia 2011; 25: 648–654. 21242993 10.1038/leu.2010.310

[14] Mezey E , Nemeth K . Mesenchymal stem cells and infectious diseases: smarter than drugs. Immunol Lett 2015; 168: 208–214. 26051681 10.1016/j.imlet.2015.05.020

[15] Olajuyin AM , Zhang X , Ji HL . Alveolar type 2 progenitor cells for lung injury repair. Cell Death Discov 2019; 5: 63. 30774991 10.1038/s41420-019-0147-9 PMC6368612

[16] Belkaid Y . Regulatory T cells and infection: a dangerous necessity. Nat Rev Immunol 2007; 7: 875–888. 17948021 10.1038/nri2189

[17] Akiyama K , Chen C , Wang D *et al* Mesenchymal‐stem‐cell‐induced immunoregulation involves FAS‐ligand‐/FAS‐mediated T cell apoptosis. Cell Stem Cell 2012; 10: 544–555. 22542159 10.1016/j.stem.2012.03.007 PMC3348385

[18] Chen ML , Yan BS , Bando Y , Kuchroo VK , Weiner HL . Latency‐associated peptide identifies a novel CD4^+^CD25^+^ regulatory T cell subset with TGF *β*‐mediated function and enhanced suppression of experimental autoimmune encephalomyelitis. J Immunol 2008; 180: 7327–7337. 18490732 10.4049/jimmunol.180.11.7327 PMC2771858

[19] Kornete M , Mason ES , Girouard J , Lafferty EI , Qureshi S , Piccirillo CA . Th1‐Like ICOS^+^ Foxp3^+^ Treg cells preferentially express CXCR3 and home to β‐islets during pre‐diabetes in BDC2.5 NOD mice. PLoS One 2015; 10: e0126311. 25946021 10.1371/journal.pone.0126311 PMC4422433

[20] Kornete M , Sgouroudis E , Piccirillo CA . ICOS‐dependent homeostasis and function of Foxp3^+^ regulatory T cells in islets of nonobese diabetic mice. J Immunol 2012; 188: 1064–1074. 22227569 10.4049/jimmunol.1101303

[21] Hasegawa H , Inoue A , Kohno M *et al* Therapeutic effect of CXCR3‐expressing regulatory T cells on liver, lung and intestinal damages in a murine acute GVHD model. Gene Ther 2008; 15: 171–182. 17989707 10.1038/sj.gt.3303051

[22] Groom JR , Luster AD . CXCR3 in T cell function. Exp Cell Res 2011; 317: 620–631. 21376175 10.1016/j.yexcr.2010.12.017 PMC3065205

[23] Muller M , Carter S , Hofer MJ , Campbell IL . Review: The chemokine receptor CXCR3 and its ligands CXCL9, CXCL10 and CXCL11 in neuroimmunity–a tale of conflict and conundrum. Neuropathol Appl Neurobiol 2010; 36: 368–387. 20487305 10.1111/j.1365-2990.2010.01089.x

[24] Mueller NJ . New immunosuppressive strategies and the risk of infection. Transpl Infect Dis 2008; 10: 379–384. 18811628 10.1111/j.1399-3062.2008.00346.x

[25] Lombardo E , van der Poll T , DelaRosa O , Dalemans W . Mesenchymal stem cells as a therapeutic tool to treat sepsis. World J Stem Cells 2015; 7: 368–379. 25815121 10.4252/wjsc.v7.i2.368 PMC4369493

[26] Hackstein H , Lippitsch A , Krug P *et al* Prospectively defined murine mesenchymal stem cells inhibit *Klebsiella pneumoniae*‐induced acute lung injury and improve pneumonia survival. Respir Res 2015; 16: 123. 26438075 10.1186/s12931-015-0288-1 PMC4594670

[27] Zhu YG , Hao Q , Monsel A , Feng XM , Lee JW . Adult stem cells for acute lung injury: remaining questions and concerns. Respirology 2013; 18: 744–756. 23578018 10.1111/resp.12093 PMC3690944

[28] Krasnodembskaya A , Song Y , Fang X *et al* Antibacterial effect of human mesenchymal stem cells is mediated in part from secretion of the antimicrobial peptide LL‐37. Stem Cells 2010; 28: 2229–2238. 20945332 10.1002/stem.544 PMC3293245

[29] Jiang J , Kelly KA . Phenotype and function of regulatory T cells in the genital tract. Curr Trends Immunol 2011; 12: 89–94. 22287829 PMC3266607

[30] Griffin MD , Elliman SJ , Cahill E , English K , Ceredig R , Ritter T . Concise review: adult mesenchymal stromal cell therapy for inflammatory diseases: how well are we joining the dots? Stem Cells 2013; 31: 2033–2041. 23766124 10.1002/stem.1452

[31] dos Santos CC , Zhang H , Slutsky AS . From bench to bedside: bacterial growth and cytokines. Crit Care 2002; 6: 4–6. 11940256 10.1186/cc1443 PMC137387

[32] Sun J , Han ZB , Liao W *et al* Intrapulmonary delivery of human umbilical cord mesenchymal stem cells attenuates acute lung injury by expanding CD4^+^CD25^+^ Forkhead Boxp3 (FOXP3)^+^ regulatory T cells and balancing anti‐ and pro‐inflammatory factors. Cell Physiol Biochem 2011; 27: 587–596. 21691076 10.1159/000329980

[33] Chao YH , Wu HP , Wu KH *et al* An increase in CD3^+^CD4^+^CD25^+^ regulatory T cells after administration of umbilical cord‐derived mesenchymal stem cells during sepsis. PLoS One 2014; 9: e110338. 25337817 10.1371/journal.pone.0110338 PMC4206342

[34] Belkaid Y , Piccirillo CA , Mendez S , Shevach EM , Sacks DL . CD4^+^CD25^+^ regulatory T cells control *Leishmania major* persistence and immunity. Nature 2002; 420: 502–507. 12466842 10.1038/nature01152

[35] Hisaeda H , Maekawa Y , Iwakawa D *et al* Escape of malaria parasites from host immunity requires CD4^+^ CD25^+^ regulatory T cells. Nat Med 2004; 10: 29–30. 14702631 10.1038/nm975

[36] Suvas S , Kumaraguru U , Pack CD , Lee S , Rouse BT . CD4^+^CD25^+^ T cells regulate virus‐specific primary and memory CD8^+^ T cell responses. J Exp Med 2003; 198: 889–901. 12975455 10.1084/jem.20030171 PMC2194203

[37] Soerens AG , Da Costa A , Lund JM . Regulatory T cells are essential to promote proper CD4 T‐cell priming upon mucosal infection. Mucosal Immunol 2016; 9: 1395–1406. 27007674 10.1038/mi.2016.19 PMC5035160

[38] Xu Z , Shi L , Wang Y *et al* Pathological findings of COVID‐19 associated with acute respiratory distress syndrome. Lancet Respir Med 2020; 8: 420–422. 32085846 10.1016/S2213-2600(20)30076-X PMC7164771

[39] Ji F , Li L , Li Z , Jin Y , Liu W . Mesenchymal stem cells as a potential treatment for critically ill patients with coronavirus disease 2019. Stem Cells Transl Med 2020; 9: 813–814. 32320535 10.1002/sctm.20-0083 PMC7264790

[40] Leng Z , Zhu R , Hou W *et al* Transplantation of ACE2^−^ mesenchymal stem cells improves the outcome of patients with COVID‐19 pneumonia. Aging Dis 2020; 11: 216–228. 32257537 10.14336/AD.2020.0228 PMC7069465

[41] Rashedi I , Gomez‐Aristizabal A , Wang XH , Viswanathan S , Keating A . TLR3 or TLR4 activation enhances mesenchymal stromal cell‐mediated Treg induction via notch signaling. Stem Cells 2017; 35: 265–275. 27571579 10.1002/stem.2485

[42] Braza F , Dirou S , Forest V *et al* Mesenchymal stem cells induce suppressive macrophages through phagocytosis in a mouse model of asthma. Stem Cells 2016; 34: 1836–1845. 26891455 10.1002/stem.2344

[43] Josefowicz SZ , Lu LF , Rudensky AY . Regulatory T cells: mechanisms of differentiation and function. Annu Rev Immunol 2012; 30: 531–564. 22224781 10.1146/annurev.immunol.25.022106.141623 PMC6066374

[44] Richard ML , Gilkeson G . Mouse models of lupus: what they tell us and what they don't. Lupus Sci Med 2018; 5: e000199. 29387435 10.1136/lupus-2016-000199 PMC5786947

[45] Zhang Z , Feng R , Niu L *et al* Human umbilical cord mesenchymal stem cells inhibit T follicular helper cell expansion through the activation of iNOS in lupus‐prone B6.MRL‐*Fas* ^lpr^ mice. Cell Transplant 2017; 26: 1031–1042. 28105982 10.3727/096368917X694660 PMC5657746

[46] Li W , Zhang X , Yang Y *et al* Recognition of conserved antigens by Th17 cells provides broad protection against pulmonary *Haemophilus influenzae* infection. Proc Natl Acad Sci USA 2018; 115: e7149–e7157. 29987031 10.1073/pnas.1802261115 PMC6065041

[47] Matute‐Bello G , Downey G , Moore BB *et al* An official American Thoracic Society workshop report: features and measurements of experimental acute lung injury in animals. Am J Respir Cell Mol Biol 2011; 44: 725–738. 21531958 10.1165/rcmb.2009-0210ST PMC7328339

[48] Rashighi M , Agarwal P , Richmond JM *et al* CXCL10 is critical for the progression and maintenance of depigmentation in a mouse model of vitiligo. Sci Transl Med 2014; 6: 223ra223. 10.1126/scitranslmed.3007811 PMC4086941 24523323

