# Supplementary material for: Mesenchymal stem cells prevent overwhelming inflammation and reduce infection severity via recruiting CXCR3+ regulatory T cells
Source: Clin Transl Immunology. 2020 Sep 30;9(10):e1181. doi: 10.1002/cti2.1181 (PMC7526004; doi:10.1002/cti2.1181)
Supplement: Supplementary file 1 — ¶ [file CTI2-9-e1181-s001.docx]

**SUPPLEMENTARY INFORMATION FOR**

Mesenchymal stem cells prevent overwhelming inflammation and reduce infection severity via recruiting CXCR3+ regulatory T cells

Wenchao Li^1#^, Weiwei Chen^1#^, Saisai Huang^1^, Genhong Yao^1^, Xiaojun Tang^1*^, Lingyun Sun^1*^

# AFFILIATION

1 Department of Rheumatology and Immunology, The Affiliated Drum Tower Hospital of Nanjing University Medical School, Nanjing, Jiangsu, 210008, China.

^#^ These authors contributed equally.

# * CORRESPONDING AUTHORS

1. Dr. Lingyun Sun, Email: lingyunsun@nju.edu.cn
2. Dr. Xiaojun Tang, Email: xjtang09@163.com

**This PDF file includes:**

1. Supplementary Methods

2. Supplementary figure 1 - 5

**Supplementary methods**

**In Vivo Staining with Anti-CD45.2.** Anti-mouse CD45.2-FITC (clone 104; BD) was diluted to 10 μg/ml in sterile PBS and 250 μl of the solution was injected *i.v.* via the tail vein 3 min before killing and sample harvest as described by Christensen *et al.*[^1^](#_ENREF_1).

**Supplementary data:**


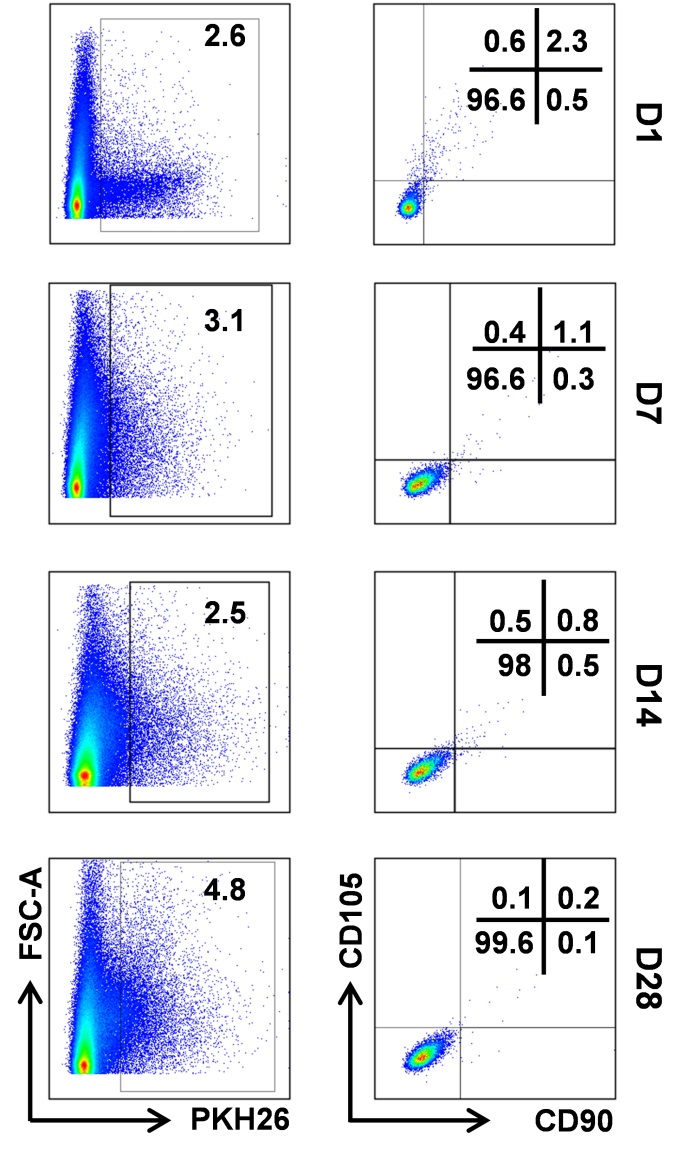


**Supplementary figure 1.** **Most MSCs were eliminated soon after intravenous infusion.** 5×10^5^ of PKH26 labelled MSCs were injected into mice intravenously PBS treated mice were set as controls. Lungs were collected at indicated time. Cells were isolated from the lungs and cells that positive for human CD105, CD90 were analysed by FACS. Representative FACS data were shown.


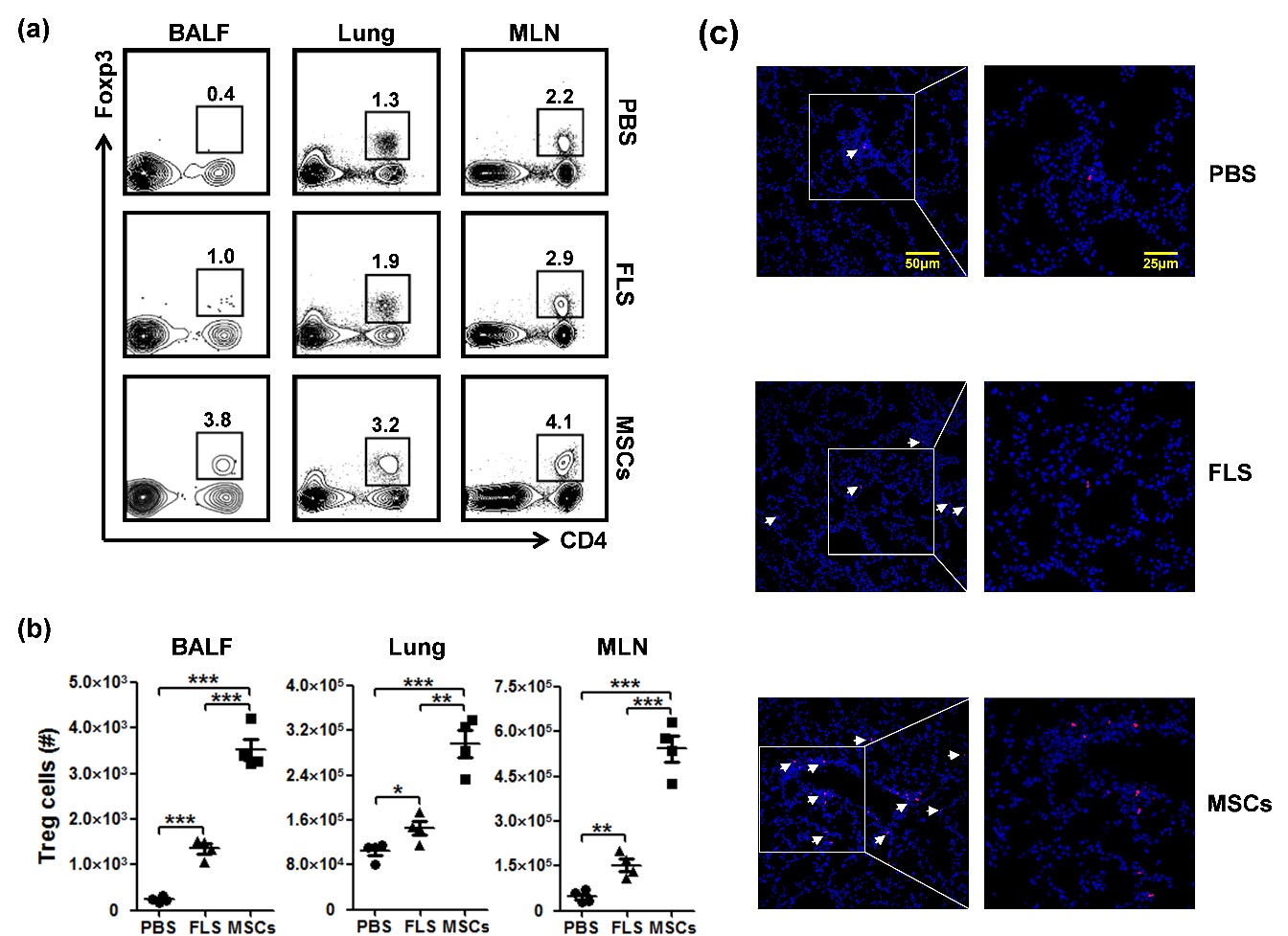


**Supplementary figure 2. Influence of MSCs on lung macrophages.** After receiving 5×10^5^ of MSCs or fibroblasts (FLS) or PBS (*i.v.*), mice were sacrificed on day 3. Tregs in the lung were determined by **(a, b)** FACS and **(c)** histoimmunofluorescence. Foxp3 (red), Nuclei (blue). Arrow head = Treg. n=4 each group, **P* < 0.05; ***P* < 0.01; ****P* < 0.001. Error bars = means ± SEM. All the experiments were repeated three times. The representative data are shown.

**Supplementary figure 3. Percentages of circulating and local Tregs in the lung and expression of CXCR3 by the two populations.** Three days after MSC transfer, mice were sacrificed. PBS-treated mice were used as controls. The percentages of circulating Tregs were determined by in vivo staining with anti-CD45.2 antibodies. Expression of CXCR3 on the lung (CD45.2^-^) and circulating (CD45.2^+^) Tregs were measured by FACS.

**
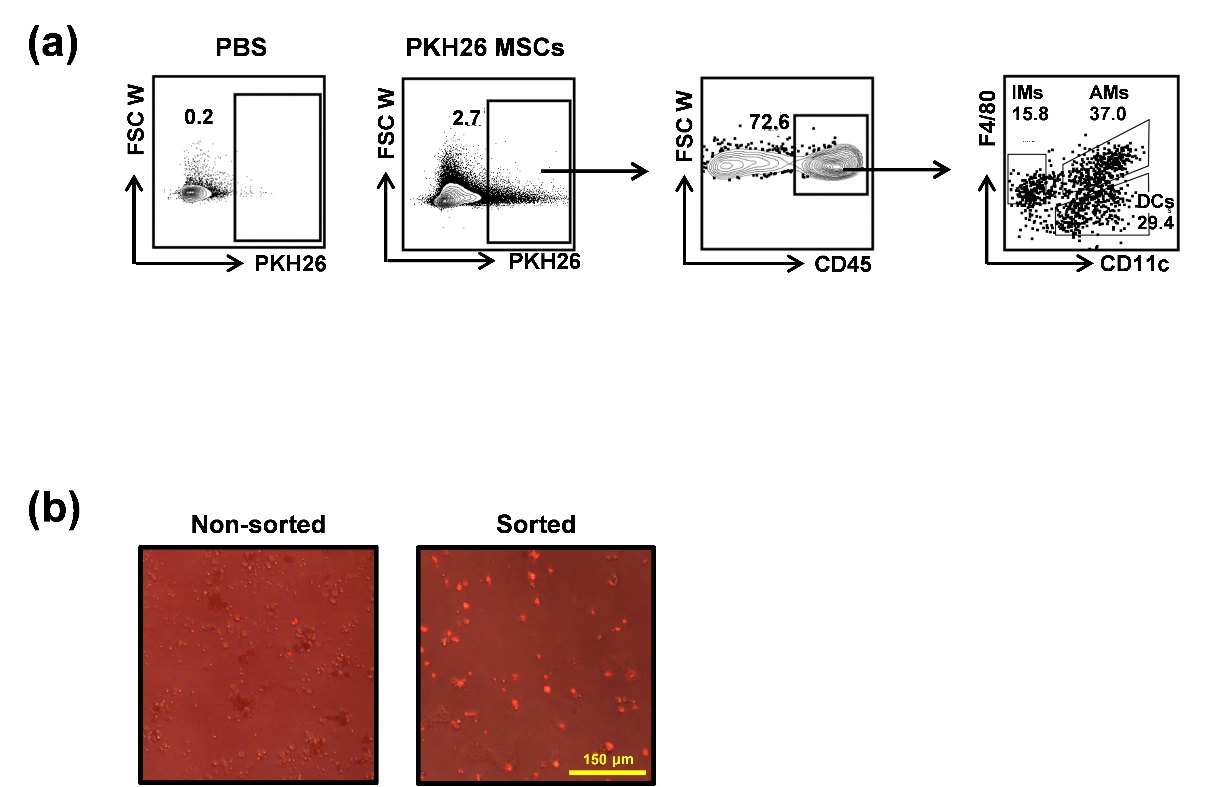
**

**Supplementary figure 4. Most MSCs were phagocytosed by lung phagocytes.** 5×10^5^ of PKH26 labelled MSCs were injected into mice intravenously PBS treated mice were set as controls. Lungs were collected on day 3 after infusion. **(a)** Cells were isolated from the lungs and the phenotype of PKH26^+^ cells were analysed by FACS. Representative FACS data were shown. **(b)** PKH26^+^CD45^+^F4/80^+^ cells were sorted. Photos were taken before and after cell sorting. The representative photos were shown.


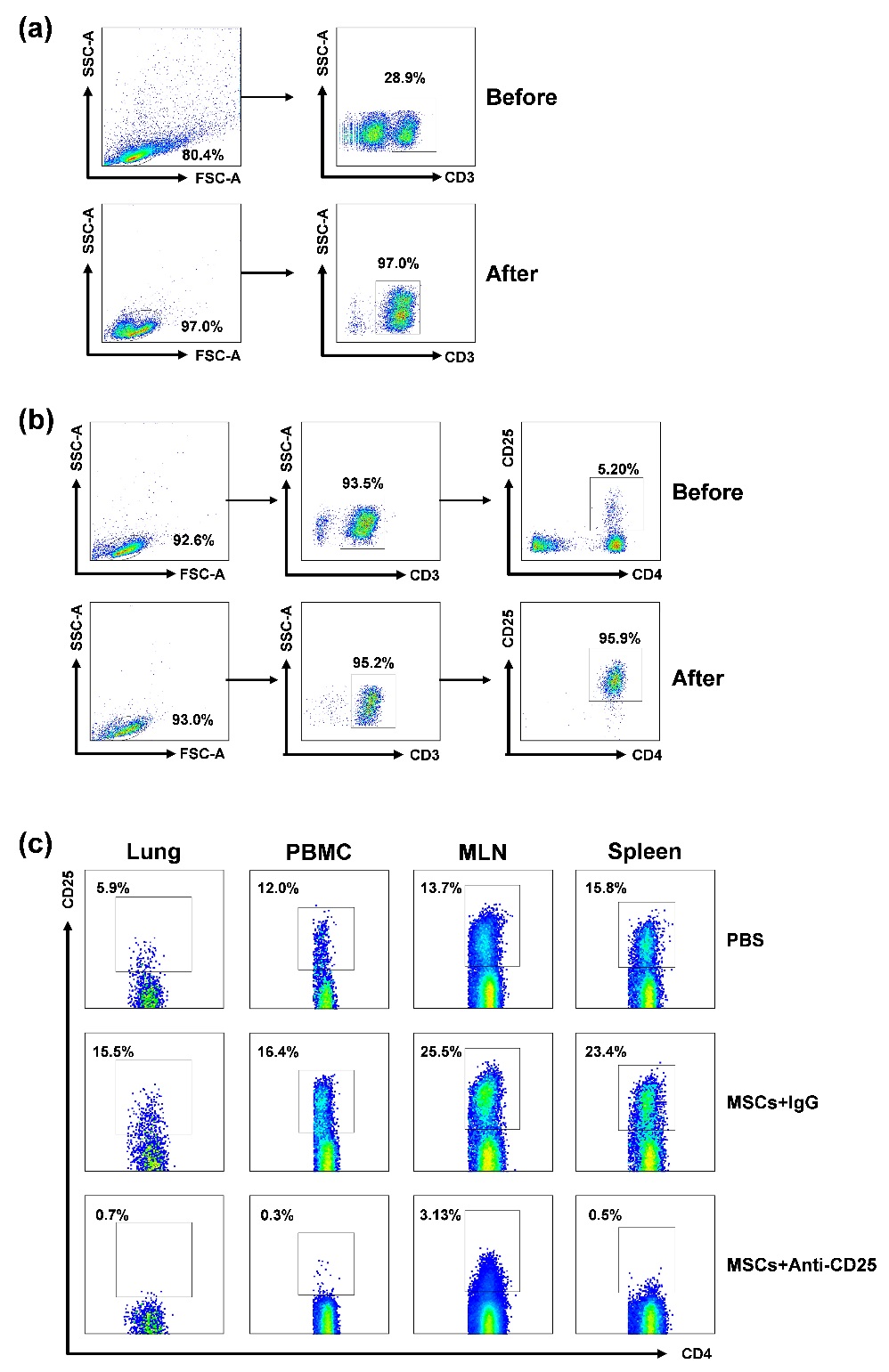


**Supplementary figure 5. Purification of Tregs and depletion of Tregs with anti-CD25 antibodies.** Three days after MSC transfer, mice were sacrificed. **(a)** Lung and spleen T cells were purified by using Pan T cell isolation Kit and the purity of the sorted cells were determined. **(b)** CD4^+^CD25^+^ cells were next sorted from the purified T cells by FACS Aria and the purity of sorted cells was detected. **(c)** After received MSCs intravenously, mice were immediately given *i.p.* injections of anti-CD25 (clone PC61) or control rat IgG antibodies. Three days after the treatments, mice were sacrificed and the frequencies of CD4^+^CD25^+^ cells in lung, PBMC, MLN and spleen were determined by FACS.

**Reference**

1 Christensen D, Mortensen R, Rosenkrands I, Dietrich J, Andersen P. Vaccine-induced Th17 cells are established as resident memory cells in the lung and promote local IgA responses. *Mucosal Immunol* 2017; **10**:260-270
